# Conservation of White Rhinoceroses Threatened by Bovine Tuberculosis, South Africa, 2016–2017

**DOI:** 10.3201/eid2412.180293

**Published:** 2018-12

**Authors:** Michele A. Miller, Peter Buss, Sven D.C. Parsons, Eduard Roos, Josephine Chileshe, Wynand J. Goosen, Louis van Schalkwyk, Lin-Mari de Klerk-Lorist, Markus Hofmeyr, Guy Hausler, Leana Rossouw, Tebogo Manamela, Emily P. Mitchell, Rob Warren, Paul van Helden

**Affiliations:** Stellenbosch University, Department of Science and Technology–National Research Foundation Centre of Excellence for Biomedical Tuberculosis Research, South African Medical Research Council Centre for Tuberculosis Research, Cape Town, South Africa (M.A. Miller, S.D.C. Parsons, E. Roos, J. Chileshe, W.J. Goosen, G. Hausler, R. Warren, P. van Helden);; South African National Parks, Skukuza, South Africa (P. Buss, M. Hofmeyr, L. Rossouw, T. Manamela);; Office of the State Veterinarian, Skukuza (L. van Schalkwyk, L.-M. de Klerk-Lorist); National Zoological Gardens of South Africa, Pretoria, South Africa (E.P. Mitchell)

**Keywords:** bovine tuberculosis, *Ceratotherium simum*, Kruger National Park, *Mycobacterium bovis*, white rhinoceros, South Africa, histology, mycobacteria, tuberculosis, granulomas, conservation, surveillance, zoonoses

## Abstract

During 2016–2017, when Kruger National Park, South Africa, was under quarantine to limit bovine tuberculosis spread, we examined 35 white and 5 black rhinoceroses for infection. We found 6 infected white rhinoceroses during times of nutritional stress. Further research on *Mycobacterium bovis* pathogenesis in white rhinoceroses is needed.

Tuberculosis (TB) caused by *Mycobacterium tuberculosis* or *M. bovis* has been reported in captive rhinoceroses since the early 1800s (*1**–**3*). Bovine TB is endemic in many wildlife populations worldwide, including among those in Kruger National Park (KNP), South Africa (*4*). KNP contains the largest free-living population of white rhinoceroses in the world (estimated at 6,649–7,830). However, prolonged drought in South Africa (2015–2017) raised concerns that starvation and disease could increase the mortality rate and affect conservation efforts for this species (*5*).

In June 2016, a black rhinoceros (*Diceros bicornis minor*) with an *M. bovis* infection was discovered (*6*). Thereafter, a surveillance program was initiated to screen rhinoceros carcasses in KNP, leading to 35 white and 5 black rhinoceros carcasses being examined during June 2016–October 2017. To determine which animals were infected, we conducted macroscopic examinations and collected samples for histopathologic studies and mycobacterial culture, as previously described (*7*). Research protocols were approved by the South African National Park Animal Use and Care Committee and ethics committee of Stellenbosch University.

No additional cases of *M. bovis* infection were found in black rhinoceroses. However, we confirmed *M. bovis* infection in 6 white rhinoceroses (Table). Grossly visible lesions, mostly found in the retropharyngeal or tracheobronchial lymph nodes or lung, were typically small and localized and could easily be missed or mistaken for granulomas caused by other pathogens if careful dissections of tissues were not performed (Technical Appendix). On histologic examination, we found granulomatous inflammation in lung or lymph node sections and rare acid-fast organisms in some granulomas (Table). We typed these *M. bovis* isolates as strain SB0121, the most common strain found in KNP (*8*).

**Table:** Findings from 6 *Mycobacterium bovis*–infected white rhinoceroses, Kruger National Park, South Africa, 2016–2017*

Case no.	Age category	Sex	Date	Body condition	Features of lesions consistent with bovine TB†			*M. bovis *culture– positive tissue pools†	AFB on cytology of lesions†
					Macroscopic, multifocal mineralized granulomas	Multifocal granulomas on histology	AFB on histology		
1	Subadult	M	Sep 2016	Thin	+ Lung and retropharyngeal LN	+ Lung and retropharyngeal LN	–	Head, thoracic, and peripheral LNs and lung	+ LNs
2	Adult	F	Sep 2016	Thin	– Lung and all LNs	ND‡	ND‡	Thoracic LN and lung	ND‡
3	Adult	M	Oct 2016	Thin	+ Lung; – all LNs	+ Lung; – LNs	–	Thoracic and abdominal LNs and lung	+ Lung
4	Subadult	F	Nov 2016	Thin	– Lung; + submandibular, retropharyngeal, tracheobronchial, and mesenteric LNs	– Lung; + submandibular, retropharyngeal, tracheobronchial, and mesenteric LNs	Rare	Head and thoracic LNs	+ LNs
5	Subadult	F	Sep 2017	Normal	+ Lung and prescapular and axillary LNs	ND	ND	Thoracic LN	+ Lung
6	Adult	M	Oct 2017	Thin	+ Lung and prescapular, retropharyngeal, and tracheobronchial LNs	ND	ND	Tracheobronchial, prescapular, and retropharyngeal LNs and lung	+ Lung and LNs

*AFB, acid-fast bacilli; LN, lymph node; ND, not done; TB, tuberculosis; + feature present; – feature absent.  †LNs were pooled into 4 sets: head (retropharyngeal, submandibular, and cervical); thoracic (tracheobronchial and mediastinal); abdominal (mesenteric and hepatic); and peripheral (axillary, prescapular, and inguinal) LNs. ‡No clinically significant lesions other than inflammation (poaching case).

Four of the infected animals were found during September–November 2016, near the end of the drought, and the remaining 2 animals were found in September and October 2017, at the end of the next winter. The timing of infections suggests that animals under nutritional stress might be more susceptible to infection, similar to observations in other species (*9*). The low number of positive cases and localized paucibacillary lesions support the hypothesis that white rhinoceroses, although susceptible to infection, are able to limit disease progression (*10*). However, whether infected animals would develop disease if compromised is unknown. Location of lesions yielding positive cultures suggests an aerosol route of exposure, although *M. bovis* was also isolated from mesenteric and peripheral lymph nodes (Table). Although no data were available to evaluate transmission, a previous study has shown white rhinoceroses with localized *M. bovis* infection did not regularly shed bacilli (*10*). Further research is required to understand the pathogenesis and epidemiology of *M. bovis* infection in these animals.

Fresh samples from animals that die naturally are difficult to locate in a large ecosystem, especially before predators arrive at the carcass or decomposition occurs due to elevated temperatures. In our study, collection of samples with minimal degradation was facilitated by our examining only rhinoceroses dead for <12 hours and animals euthanized because of their severe state of debilitation, most often from poaching wounds. Bovine TB was not considered the cause of the poor condition or death in any of these animals. We found small, nonspecific culture-positive lesions histologically similar to those caused by helminths, foreign material, and fungi, and the paucibacillary nature of the infection could result in false-negative histopathologic results. Therefore, we needed to confirm infection by mycobacterial culture and species determination with every tissue set collected. However, low numbers of viable bacteria, sample handling, and the likelihood of overgrowth by contaminants could also lead to false-negative culture results. Positive culture results from >1 tissue sample in the same rhinoceros suggests infection rather than contamination. However, no cases of disseminated bovine TB have been observed in this species, supporting the authors’ hypothesis that the disease in white rhinoceroses is self-limiting. Factors such as drought might play a role in altering susceptibility to infection, considering no positive culture results were obtained in >20 rhinoceros carcasses examined before June 2016.

Although disease and death associated with bovine TB have not been observed in white rhinoceroses, *M. bovis* infection nonetheless presents a threat to conservation of this species. Genetic management and translocation of rhinoceroses are essential components of in situ conservation; animals need to be moved from high-risk poaching areas to more secure locations (*5*). In addition, calves orphaned by poaching require intensive specialized care, which is only available outside KNP (*5*). Because *M. bovis* is a controlled disease, premises with infected populations are placed under quarantine to prevent translocation of potentially infected animals. With a paucity of data to assess risks, movement restrictions are a substantial impediment to conservation and can threaten the survival of this population. Therefore, research into antemortem detection, pathogenesis, and epidemiology of *M. bovis* infection is essential for programs to conserve rhinoceroses of Africa.

Technical Appendix Description of methods and photographs of lung and retropharyngeal lymph node of *Mycobacterium bovis*–infected white rhinoceroses.
