# Supplementary material for: Conservation of White Rhinoceroses Threatened by Bovine Tuberculosis, South Africa, 2016–2017
Source: Emerg Infect Dis. 2018 Dec;24(12):2373–5. doi: 10.3201/eid2412.180293 (PMC6256411; doi:10.3201/eid2412.180293)
Supplement: Technical Appendix — Description of methods and photographs of lung and retropharyngeal lymph node of Mycobacterium bovis–infected white rhinoceroses. [file 18-0293-Techapp-s1.pdf]

Article DOI: https://doi.org/10.3201/eid2412.180293

Conservation of White Rhinoceroses Threatened by Bovine Tuberculosis, South Africa, 2016–2017

Technical Appendix

Heparinized whole blood collected before death was used to assess interferon γ (IFN-γ) release in response to mycobacteria-specific antigenic peptides by using a modified QuantiFERON TB Gold (In-Tube) (QIAGEN, Venlo, Limburg, the Netherlands) assay as previously described (*1*,*2*). The commercial Bovine IFN-γ ELISA development kit (Mabtech AB, Nacka Strand, Sweden), which is cross-reactive with IFN-γ of sheep and horses, was used in combination with a bovine recombinant IFN-γ standard curve to measure rhinoceros IFN-γ. Rhinoceros IFN-γ has been previously detected in white rhinoceroses experimentally infected with *Mycobacterium bovis* (*2*), and a cutoff value of 36 pg/mL was determined by using QuantiFERON-TB–stimulated whole blood from uninfected white rhinoceroses (M.A. Miller, unpub. data). Five rhinoceroses had detectable antigen-specific IFN-γ responses of 5, 20, 24, 75, and 125 pg/mL, which suggests immune sensitization to *M. tuberculosis* complex in at least 2 animals (those with the higher IFN-γ values). The low level of immune sensitization supports the hypothesis that white rhinoceroses have limited infections with *M. bovis* (*1*).

Detailed macroscopic examinations were conducted with lung and numerous lymph nodes: head (submandibular, retropharyngeal, and cervical); thoracic (tracheobronchial and mediastinal); abdominal (mesenteric and hepatic); and peripheral (prescapular, axillary, and inguinal). Representative samples were frozen for mycobacterial culture and preserved in 10% buffered formalin for histopathology. Tissues were processed and inoculated for culture by using the BACTEC MGIT Mycobacterial Growth Indicator Tube system (Becton Dickinson, Franklin Lakes, NJ, USA), as previously described (*2*). For all positive cultures, the species was determined by PCR of regions of difference (*3*) and spoligotyping (*4*).

Gross lesions were typically localized and varied from consolidated tissue and single pinpoint lesions to multifocal, small, mineralized granulomas (Technical Appendix Figures 1, 2). The location of lesions and the tissues that were culture positive for *M. bovis* in each

animal are shown in main text Table. All 6 white rhinoceroses had *M. bovis* isolated from thoracic (tracheobronchial, mediastinal, or both) lymph node tissue cultures, and 4 animals had positive cultures from lung tissue, suggesting inhalation as the route of exposure (main text Table). Culture results were concordant with the histologic presence of acid-fast organisms in only 1 animal (out of 3 with histologic granulomas), although cytologic evaluation revealed acid-fast organisms in all 5 rhinoceroses in which direct lesion smears were performed.

Histopathologic findings in 3 of 4 cases examined included the following. In case 1, moderate multifocal chronic granulomatous lymphadenitis was present in the retropharyngeal lymph nodes, characterized by large discrete foci of mineralized necrotic tissue surrounded by a thin layer of macrophages, epithelioid cells, and rare multinucleate giant cells, which were encapsulated by a thick layer of fibrous connective tissue containing small-to-moderate numbers of lymphocytes and plasma cells. This rhinoceros also had mild, multifocal chronic granulomatous pneumonia with an appearance similar to lymph nodes, as well as some fibrous foci and foci with nonmineralized necrotic debris. In case 3, the lungs contained multiple small granulomas ranging from encapsulated lymphoplasmacytic and histiocytic inflammation surrounding necrotic foci to discrete fibrous foci of mineralized necrotic tissue, minimal inflammation, and discrete fibrous foci with no associated inflammation or mineralization. Case 4 had the most substantial microscopic changes of the 3 cases. We found severe multifocal chronic necrogranulomatous lymphadenitis characterized by multiple pale-tan, discrete, and coalescing foci in several lymph nodes (<7 x 5 mm) consisting of foci of variably mineralized lytic necrotic tissue debris, surrounded by thin layers of macrophages, multinucleate giant cells, and epithelioid cells and a variability thick capsule of immature fibrous connective tissue.

References

1. Michel AL, Lane EP, de Klerk-Lorist L-M, Hofmeyr M, van der Heijden EMDL, Botha L, et al. Experimental *Mycobacterium bovis* infection in three white rhinoceroses (*Ceratotherium simum*): Susceptibility, clinical and anatomical pathology. PLoS One. 2017;12:e0179943. PubMed http://dx.doi.org/10.1371/journal.pone.0179943

2. Parsons SDC, Morar-Leather D, Buss P, Hofmeyr J, McFadyen R, Rutten VPMG, et al. The kinetics of the humoral and interferon-gamma immune responses to experimental *Mycobacterium bovis* infection in the white rhinoceros (*Ceratotherium simum*). Front Immunol. 2017;8:1831. PubMed http://dx.doi.org/10.3389/fimmu.2017.01831

3. Goosen WJ, Miller MA, Chegou NN, Cooper D, Warren RM, van Helden PD, et al. Agreement between assays of cell-mediated immunity utilizing *Mycobacterium bovis*-specific antigens for the diagnosis of tuberculosis in African buffaloes (*Syncerus caffer*). Vet Immunol Immunopathol. 2014;160:133–8. PubMed http://dx.doi.org/10.1016/j.vetimm.2014.03.015

4. Warren RM, Gey van Pittius NC, Barnard M, Hesseling A, Engelke E, de Kock M, et al. Differentiation of *Mycobacterium tuberculosis* complex by PCR amplification of genomic regions of difference. Int J Tuberc Lung Dis. 2006;10:818–22. PubMed

5. Kamerbeek J, Schouls L, Kolk A, van Agterveld M, van Soolingen D, Kuijper S, et al. Simultaneous detection and strain differentiation of *Mycobacterium tuberculosis* for diagnosis and epidemiology. J Clin Microbiol. 1997;35:907–14. PubMed

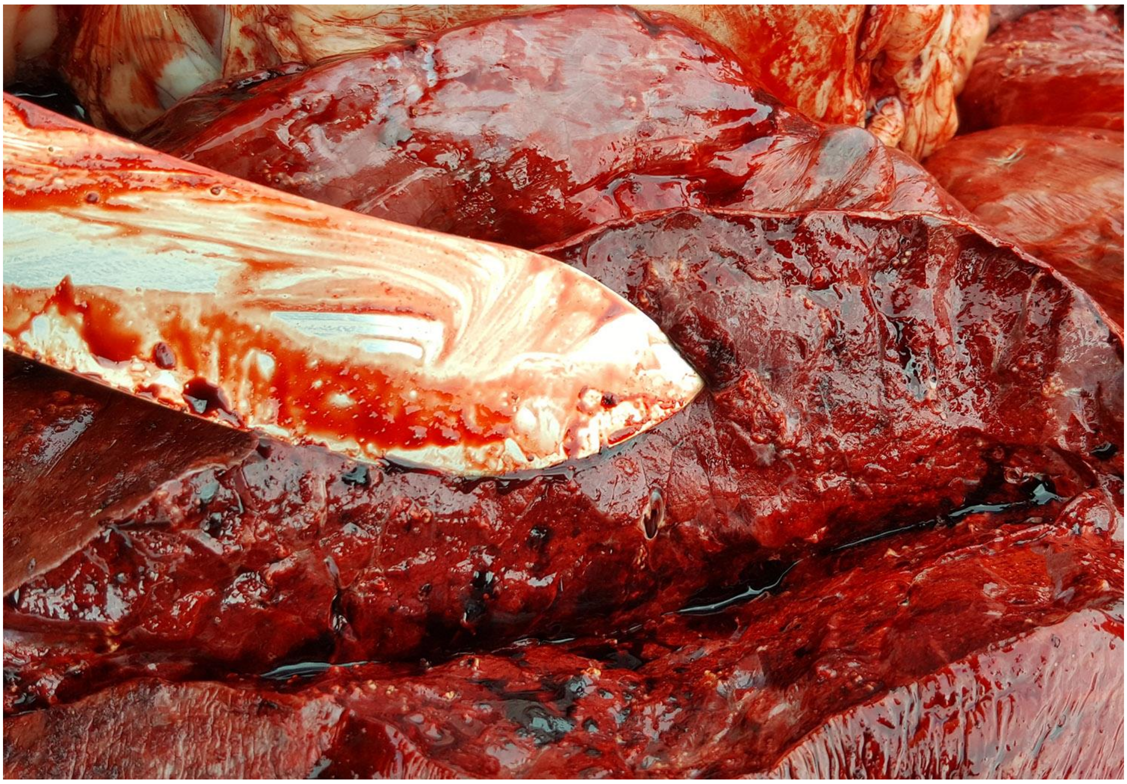

Technical Appendix Figure 1. Area of consolidation in white rhinoceros lung tissue showing a nonspecific lesion from which *Mycobacterium bovis* was isolated.

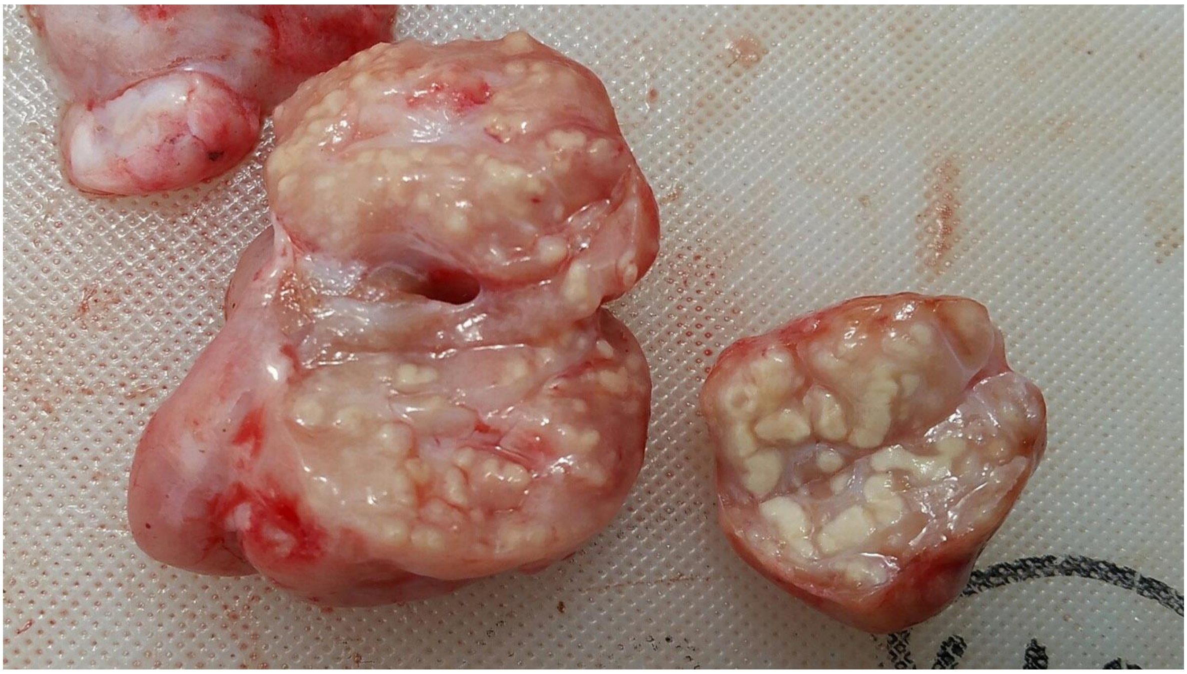

Technical Appendix Figure 2. Multifocal granulomas in the retropharyngeal lymph node of a white rhinoceros with confirmed *Mycobacterium bovis* infection.
